# Editorial for Special Issue “Genomics Approaches in Microbial Ecology”

**DOI:** 10.3390/microorganisms14030534

**Published:** 2026-02-25

**Authors:** Hugo César Ramírez-Saad, César Hugo Hernández-Rodríguez

**Affiliations:** 1 Departamento Sistemas Biológicos, Universidad Autónoma Metropolitana-Xochimilco, Mexico City 04960, Mexico; 2 Departamento de Microbiología, Escuela Nacional de Ciencias Biológicas, Instituto Politécnico Nacional, Mexico City 11430, Mexico

Microbial ecology has entered a phase of methodological consolidation where genomic and molecular tools are being used to address ecological questions rather than being subordinate to culture-based approaches. New bioinformatic tools and advances in sequencing methodologies, genome assembly and reconstruction have enabled researchers to move beyond descriptive community reports toward mechanistic interpretations of microbial function, adaptation, and interaction across environments. The articles assembled in this Microorganisms Special Issue “Genomics Approaches in Microbial Ecology” (freely available at www.mdpi.com/journal/microorganisms/special_issues/AC5511QTUW, accessed on 16 January 2026) collectively showcase how diverse gene- and genome-centered methodologies are currently being applied to address ecological inquiries in a range of environments, including aquatic and soil systems, extreme environments, host-associated systems and anthropogenic niches.

The central methodological theme across all the contributions is the integration of high-throughput sequencing with ecologically informed experimental design. Amplicon-based profiling of conserved taxonomic markers (16S rRNA gene and fungal ITS regions) remains an essential initial step for assessing community structure, enrichment dynamics, and phylogenetic placement. However, these studies go beyond descriptive surveys by using marker-gene data as a scaffold for further genomic analyses that address ecological function, niche specialization, and evolutionary relationships (Rakitin et al., 2024; Esposito et al., 2025; Ruzaini et al., 2026).

Within this collection, whole-genome sequencing and genome-resolved metagenomics emerge as core tools for microbial ecology. Several studies employ Illumina short-read sequencing, often complemented by long-read platforms such as Oxford Nanopore, to reconstruct high-quality genomes and metagenome-assembled genomes (MAGs). These hybrid sequencing strategies are particularly effective in complex or difficult-to-cultivate systems, enabling the recovery of nearly complete or closed genomes from environmental samples. Such approaches facilitate robust analyses of gene content, metabolic pathways, mobile genetic elements, and genome architecture being key components for interpreting microbial ecological strategies (Salinas-Virgen et al., 2024; Rakitin et al., 2024).

The Special Issue also highlights the continued relevance of molecular typing and comparative genomics in ecological contexts. Multilocus sequence typing (MLST), multilocus variable-number tandem-repeat analysis (MLVA), and average nucleotide identity (ANI) metrics are applied not only for taxonomic resolution but also for exploration of the population structure, strain-level diversity, and ecological differentiation. When embedded within genome-wide analyses, these methods provide a scalable framework for linking genetic variability to environmental distribution and adaptive potential (Wu et al., 2024; Man et al., 2024).

A particularly prominent methodological advance is the application of pan-genomic frameworks. By integrating multiple genomes across taxa or ecological niches, pan-genomics enables the identification of core, accessory, and unique gene sets, offering insights into functional redundancy, metabolic versatility, and evolutionary trajectories within microbial populations and communities. When coupled with curated annotation pipelines and pathway-level analyses, pan-genomic approaches move microbial ecology toward a predictive, system-level discipline (Shi et al., 2025).

This collection of papers also highlights the relevance of targeted gene and functional analyses of biosynthetic gene clusters (BGCs) and metabolite-linked features (such as antifungals), further complemented by genome mining. These approaches have enabled the elucidation of chromosomal rearrangements within closely related *Streptomyces* strains, resulting in a vast variety of secondary metabolites and antifungal biosynthetic clusters. When interpreted within a genomic framework, these targeted methodologies bridge molecular function to its ecological and physiological relevance (González-Silva et al., 2024).

Together, the studies in this Special Issue demonstrate how genomic approaches have become essential for modern microbial ecology, enabling researchers to interrogate microbial systems across different organization levels, from genes and genomes to populations and communities. By highlighting a variety of complementary molecular methodologies, this collection provides a roadmap for future research aimed at deciphering the dynamics, resilience, and function of microbes in an increasingly complex and changing world.

